# PiwiRNA-651 as marker of treatment response and survival in classical Hodgkin lymphoma

**DOI:** 10.18632/oncotarget.10015

**Published:** 2016-06-14

**Authors:** Anna Cordeiro, Alfons Navarro, Anna Gaya, Marina Díaz-Beyá, Blanca Gonzalez-Farré, Joan Josep Castellano, Dolors Fuster, Carmen Martínez, Antonio Martínez, Mariano Monzó

**Affiliations:** ^1^ Molecular Oncology and Embryology Laboratory, Human Anatomy Unit, School of Medicine, University of Barcelona, IDIBAPS, Barcelona, Spain; ^2^ Hematology Department, Hospital Clinic Barcelona, IDIBAPS, Barcelona, Spain; ^3^ Hematopathology Section, Hospital Clinic Barcelona, IDIBAPS, Barcelona, Spain

**Keywords:** Hodgkin lymphoma, non-coding RNAs, piwiRNAs, piR-651, Piwi proteins

## Abstract

PiwiRNAs, small non-coding RNAs processed by Piwi proteins, are involved in maintaining genome stability in germline cells. Recently, piwiRNA expression has been identified in some tumors. We have examined the potential reactivation of the Piwi/piwiRNA pathway in classical Hodgkin lymphoma (cHL). We found that Piwi proteins and three selected piwiRNAs, including piR-651, were expressed in cHL patients and cell lines, indicating that the Piwi/piwiRNA pathway is active in cHL. Interestingly, low levels of piR-651 were associated with lack of complete response to first-line treatment, as well as shorter disease-free and overall survival in a cohort of 94 cHL patients. At diagnosis, piR-651 was underexpressed in cHL serum samples compared to healthy controls, while after complete remission, piR-651 levels increased to levels similar to healthy controls. This is the first evidence that piwiRNAs are active in tumor and serum samples and impact prognosis in cHL.

## INTRODUCTION

Classical Hodgkin lymphoma (cHL) comprises 11% of all lymphomas and is characterized by the presence of few tumoral cells, the so called Hodgkin Reed-Sternberg (HRS) cells, surrounded by a reactive microenvironment [1]. In cHL lymph nodes, the tumor bulk is mostly composed of T (CD4+ and cytotoxic T cells) and B cells, and macrophages and other cell types that crosstalk with the HRS cells through characteristic surface molecules expressed in both HRS cells and reactive cells and through secreted cytokyines and chemokines [2]. Most cHL patients can be cured using current treatment strategies. However, about 20% of the patients will die after relapse or progressive disease, indicating a need to identify prognostic markers that improve the International Prognostic score (IPS) [3, 4]. Several approaches to extend our knowledge of cHL biology and to identify prognostic biomarkers have been addressed, including the study of purified HRS cells [5], the study of the complete lymph node [6, 7] and the study of circulating biomarkers [8]. Recently, expression profiling of the complete lymph node identified a tumor-associated macrophage signature associated with outcome in cHL patients [9], demonstrating that the analysis of both tumor cells and the microenvironment can be an effective approach to understanding the behavior of cHL patients [10].

Non-coding RNAs have recently emerged as useful biomarkers, especially the small non-coding RNAs that are involved in post-transcriptional regulation mediated by Argonaute proteins. Argonaute proteins consist of two families: the Argonaute subfamily, which binds to microRNAs and siRNAs and the Piwi subfamily which binds exclusively to piwiRNAs (piRNAs) [11]. piRNAs are small non-coding RNAs (26-32nt), first discovered in 2006 simultaneously by five groups as being expressed exclusively in mammalian testes [12–16]. piRNAs are generated by two different biogenesis pathways, known as the primary and secondary pathways (Supplementary Figure S1). In the primary pathway, after transcription the piRNA precursors are processed and transported to the cytoplasm where they are finally loaded onto Piwil1 and Piwil2 proteins. In the secondary pathway (also known as the “ping-pong” cycle), Piwil2 and Piwil4 bind in different steps to piRNAs generated by the primary pathway. During the ping-pong cycle, the piRNA binds by sequence complementarity to a retrotransposon, producing at the end of the cycle the degradation of the target and a new copy of the piRNA [17]. This pathway, which depends on the abundance of both the specific piRNA and its target, leads to a selective amplification of piRNAs derived from active transposons. According to the latest update on piRNABank (2015) [18], a database which provides comprehensive information on piRNAs, more than 23,000 piRNAs have been identified in humans. In adults, most of these piRNAs are only expressed in germline tissues, where they are crucial for fertility, as evidenced by the spermatogenesis defects observed in mice knockouts for Piwi proteins [19, 20]. In the germline, the key function of piRNAs is the repression of transposons, in order to prevent mutations caused by these mobile elements [21]. They regulate transposons at different levels, including the degradation of RNA [21] through the secondary pathway and the regulation of transposon expression by DNA methylation or histone modification [22]. Other functions have also been attributed to piRNAs, including the post-transcriptional regulation of mRNAs [23].

Recently, piRNAs have been detected outside of germinal tissues [24], and interestingly, some of them are deregulated in tumor tissues. To date, only a few studies have analyzed the role of piRNAs in tumorogenesis and few piRNAs have been identified. piR-651, piR-4987, piR-20365, piR-20489, piR-20582, piR-932, piR-Hep1, and piR-823 have been shown to be upregulated in several tumors [25–28]; while only piR-823 has been described as downregulated [29]. However, the specific prognostic impact of piRNAs remains to be elucidated.

In the present work, we have investigated if the Piwi/piRNA pathway is active in cHL by studying the expression of Piwi proteins and the expression of selected piRNAs: piR-651, piR-20365 and piR-20582 [25, 26, 30]. We have found that the Piwi/piRNA pathway is indeed active in cHL and that the study of piRNAs can be a good source of prognostic markers.

## RESULTS

### Piwi/piRNA pathway is active in cHL

To investigate if the Piwi/piRNA pathway was active in cHL, we analyzed the expression of *PIWIL1*, *PIWIL2* and *PIWIL4* and that of piR-651, piR-20365 and piR-20582 [25, 26, 30].

**Figure 1:**
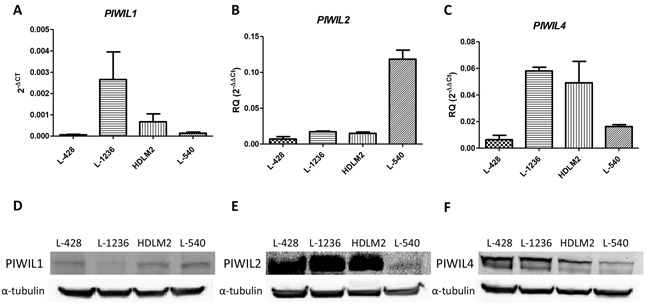
*PIWIL1*, *PIWIL2* and *PIWIL4* expression in cHL *PIWIL1* **A.** *PIWIL2* **B.** and *PIWIL4* **C.** mRNA expression in four cHL cell lines. PIWIL2-4 were showed as RQ calibrated with the expression of peripheral blood B-cells, while PIWIL1 was showed in 2^−ΔCt^ since it is not expressed in B-cells. The graph shows the median and SEM of three technical replicates. Western blot for PIWIL1 **D.** PIWIL2 **E.** and PIWIL4 **F.** in HL cell lines.

*PIWIL1*, *PIWIL2* and *PIWIL4* showed different patterns of expression in the four cHL cell lines. *PIWIL1* was mostly expressed in L-1236 and HDLM2 (Figure 1A), while *PIWIL2* and *PIWIL4* were expressed in all four cell lines (Figure 1B–1C). When we compared the expression in the cell lines with those in B-cells from peripheral blood from healthy controls, we observed that PIWIL2 and PIWIL4 were downregulated in the HL cell lines, but PIWIL1 was not expressed in the B-cells.

**Figure 2:**
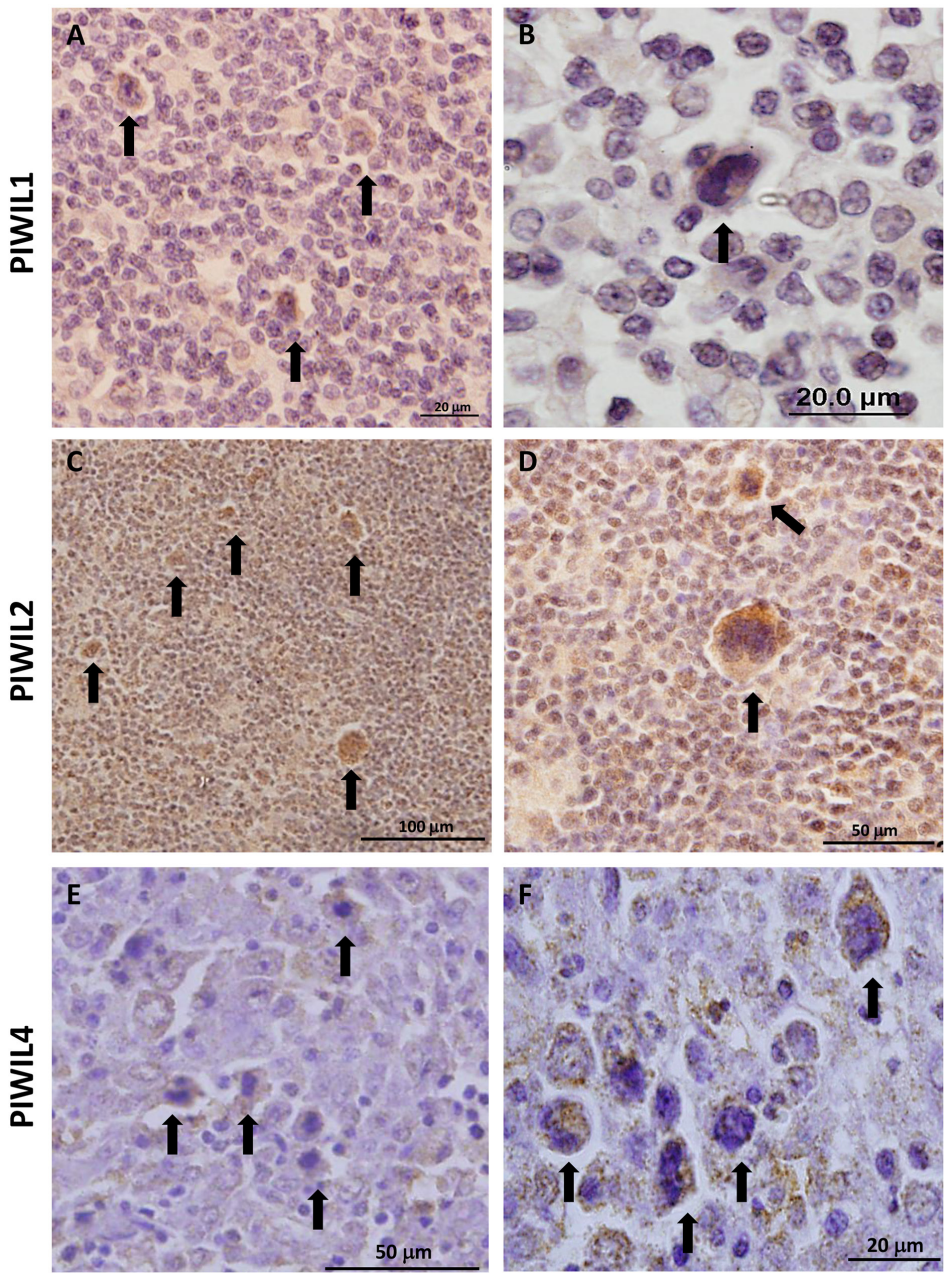
Immunohistochemistry of protein expression of Piwil1 **A, B.** Piwil2 **C, D.** and Piwil4 **E, F.** in patient lymph nodes. Arrows indicate representative HRS cells.

The immunohistochemical analysis of cHL patient samples showed that Piwil1 was expressed in only 5 of 15 cases, exclusively in the cytoplasm of the HRS cells (Figure 2A–2B). In contrast, Piwil2 was detected in all the samples, both in the HRS cells and in the reactive microenvironment (Figure 2C–2D). Finally, Piwil4 was detected in 11 of 15 cases, only in the cytoplasm of the HRS cells (Figure 2E–2F).

**Figure 3:**
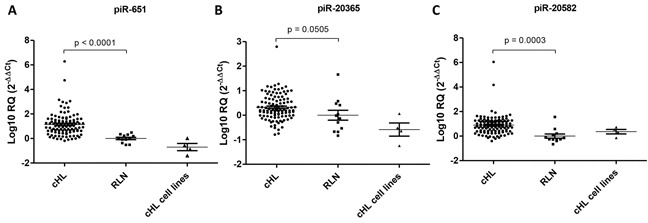
Expression levels of three selected piRNAs Expression levels of piR-651 **A.** piR-20365 **B.** and piR-20582 **C.** in 94 cHL lymph nodes, 12 reactive lymph nodes (RLN) and four cHL cell lines.

All three piRNAs were expressed in all four cell lines and in all tumor samples (Figure 3). Moreover, piR-651 (p<0.0001) and piR-20582 (p=0.0003) were significantly upregulated in patient lymph nodes compared to RLN, while piR-20365 (p=0.0505) showed a trend to upregulation (Figure 3A–3C).

### piR-651 and clinical outcome

**Figure 4:**
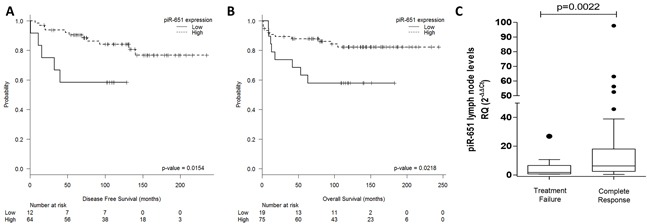
PiR-651 impacts outcome in cHL patients Disease-free survival **A.** and overall survival **B.** according to piR-651 levels. **C.** piR-651 levels according to response to first-line treatment (Treatment failure vs Complete response).

Only piR-651 expression was associated with clinical outcome. Expression levels were dichotomized using the cutoff identified by MaxStat (cutoff=0.25; mean expression of low group:-0.046 [range:-0.37-0.18]; mean expression of high group: 1.27 [range: 0.26-6.11]). Low expression levels of piR-651 were associated with shorter DFS (Mean DFS: 83.3 vs. 197.9 months, p=0.0154) and shorter OS (Mean OS: 117.2 vs. 207.2 months; p=0.0218) (Figure 4A–4B). In the multivariate analysis, including all the individual clinical factors included in the International Prognostic Score(IPS) [3], low piR-651 emerged as an independent prognostic factor for DFS (OR, 4.21; 95% CI=1.342-13.209; p=0.014) and OS (OR, 2.836; 95% CI=1.042-7.717; p=0.041) (Table 1).

**Table 1:** Multivariate analyses

Disease-Free Survival	Odds Ratio (95% CI)	*P*
Male sex	2.855 (0.887-9.187)	0.078
Age>45 years	2.478 (0.579-10.612)	0.221
Stage III-IV	1.320 (0.324-5.381)	0.699
Hemoglobin <105 g/L	8.458 (0.974-73.450)	0.053
**Albumin <40g/L**	**3.274 (1.058-10.136)**	**0.040**
White-cell count >15,000/mm3	2.113 (0.413-10.808)	0.369
Lymphocyte count <600/mm^3^ or <8%	<0.001 (<0.001-<0.001)	0.986
**Low piR-651 expression**	**4.210 (1.342-13.209)**	**0.014**

Overall Survival	Odds Ratio (95% CI)	*P*
Male sex	1.535 (0.564-4.177)	0.402
**Age>45 years**	**13.681 (4.281-43.726)**	**<0.0001**
Stage III-IV	2.107 (0.583-7.620)	0.256
Hemoglobin <105 g/L	0.820 (0.259-2.598)	0.736
Albumin <40g/L	2.828 (0.996-8.032)	0.051
**White-cell count > 15,000/mm3**	**4.282 (1.332-13.762)**	**0.015**
Lymphocyte count <600/mm3 or <8%	2.782 (0.810-9.553)	0.104
**Low piR-651 expression**	**2.836 (1.042-7.717)**	**0.041**

In the subset of 56 advanced-stage patients, piR-651 retained its impact on both DFS (p=0.003) and OS (p=0.007), while the IPS was significant only in OS (p=0.04). In the multivariate analyses including the IPS score and piR-651, piR-651 emerged as an independent prognostic factor for both DFS (OR, 6.52; 95% CI=1.72-24.77; p=0.006) and OS (OR, 2.92; 95% CI=1.07-7.93; p=0.036), while IPS was identified as a prognostic factor for OS (OR, 3.1; 95% CI=1.13-8.5; p=0.028).

### piR-651 is associated with complete response

When patients were classified as responders or non-responders to first-line treatment, non-responders had lower levels of piR-651 expression in lymph nodes (T-test p=0.0022) (Figure 4C). Using ROC curves to determine the capacity of piR-651 to distinguish responders vs non-responders to first line treatment, we found that piR-651 significantly discriminates between responders and non-responders (AUC: 0.741, 95% CI 0.594-0.888; p=0.010). When piR-651 expression was analyzed as a dichotomous variable (cutoff: 0.25; sensitivity: 0.84; 1-specificity: 0.45), lower levels of piR-651 were also associated with treatment failure. Only 7.2% of patients with high piR-651 expression had treatment failure, compared to 66.7% of those with low expression (Fisher's Exact test, p=0.009).

Moreover, we performed a multivariate analysis including Age (≤45 vs >45), number of cycles of chemotherapy (≤4 vs >4), radiotherapy (Yes vs No), stage (I-II vs III-IV) and piR-651 (Low vs High). Low piR-651 emerged as an independent prognostic factor for treatment failure (OR, 11.680; 95% CI=1.718-79.423; p=0.012) together with the ≤4 cycles of chemotherapy administered (OR, 11.761; 95% CI=1.497-92.406; p=0.019).

### piR-651 levels in serum samples

**Figure 5:**
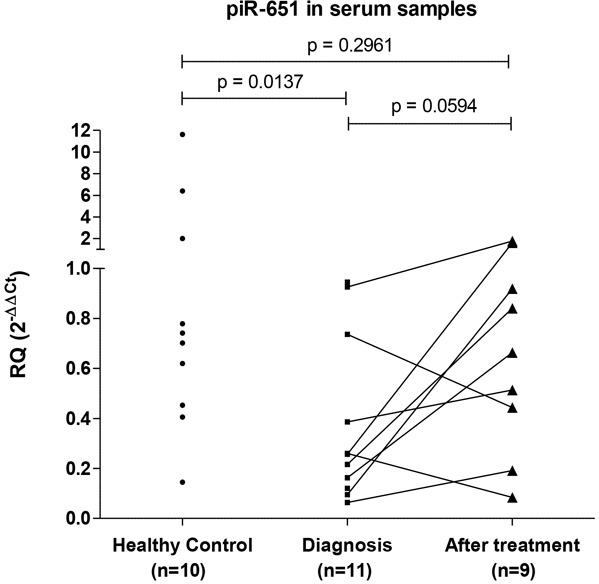
PiR-651 levels in serum samples PiR-651 was studied in serum samples from healthy controls and from cHL patients prospectively collected. From cHL patients paired samples at diagnosis and at CR after first line treatment were included.

When we studied piR-651 expression in prospectively collected serum samples, we observed that at diagnosis piR-651 was underexpressed in cHL samples (n=11) compared to serum from healthy controls (n=10) (p=0.0137). Interestingly, after CR (n=9), piR-651 showed a trend to upregulation in serum in comparison to diagnostic samples (p=0.0594), reaching levels similar to those of healthy controls. At CR, no significant differences between patients and healthy controls were observed (p=0.296) (Figure 5).

### piR-651 is expressed in HRS cells

**Figure 6:**
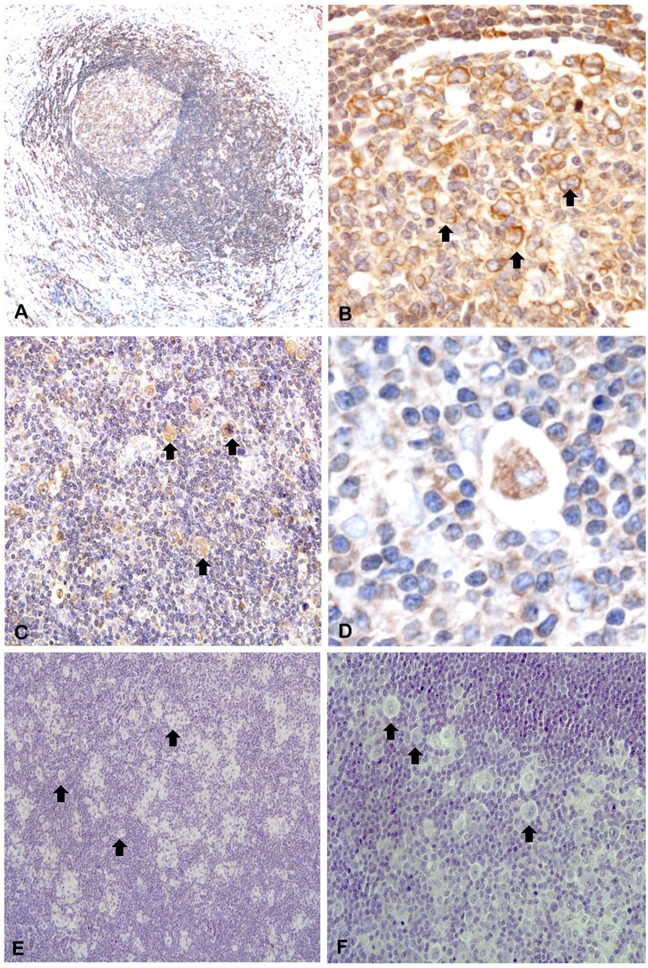
*In situ* hybridization of piR-651 Arrows indicate representative HRS cells. **A.** piR-651 was expressed in normal follicles at low levels in the mantle cells and **B.** overexpressed in large centroblasts in the reactive germinal centers. **C-D.** piR-651 was highly expressed in the cytoplasm of HRS cells. **E-F.** negative control.

To verify that the prognostic role of piR-651 was associated with its expression in HRS cells, we performed *in situ* hybridization analysis in lymph node tissue sections of four patients. piR-651 was expressed in normal follicles (Figure 6A) at low levels in the mantle cells and overexpressed in large centroblasts in the reactive germinal centers (Figure 6B). Moreover, piR-651 was highly expressed in the cytoplasm of HRS cells (Figure 6C–6D).

## DISCUSSION

Small non-coding RNAs have emerged as important post-transcriptional regulators, especially siRNAs and microRNAs [31]. However, piRNAs have been understudied because until recently they were thought to be expressed exclusively in germinal tissues and early embryonic development and silenced in differentiated tissues [32]. However, recent findings on the detection of piRNAs in several tumors have shed light on the importance of this forgotten small non-coding RNA group [30, 33]. In the present work, we have investigated whether the Piwi/piRNA pathway could be involved in the tumorogenesis process in cHL. We first studied *PIWIL1*, *PIWIL2* and *PIWIL4*, which bind specifically to piRNAs and participate in their biogenesis [34]. The study of these genes in cHL cell lines showed two different expression patterns. *PIWIL1* expression levels were very low in comparison to those of *PIWIL2* and *PIWIL4*. Moreover, *PIWIL1* expression was only detected at significant levels in one of four cHL cell lines. In contrast, *PIWIL2* and *PIWIL4* were expressed in all four cHL cell lines. This difference may be due to their participation in the two different biogenesis pathways [35]. *PIWIL1* acts exclusively in the primary biogenesis pathway, while *PIWIL4* is active only in the secondary pathway and *PIWIL2* acts in both. The differences in *PIWIL1* expression in the different cell lines may thus indicate that the primary pathway is not always active. The primary pathway is in charge of generating new piRNA sequences, while the secondary pathway helps to maintain the existent piRNA pool in the cell [35]. Interestingly, in several solid tumors, the reactivation of *PIWIL1* expression, associated with worse prognosis, could be related to a stem cell phenotype [36–39]. Similarly, *PIWIL1* is expressed in CD34+ hematopoietic stem cells [40]. When we studied Piwi protein expression in patient lymph nodes, we observed a similar pattern to that in cell lines. Piwil1 was only detected in the cytoplasm of tumor cells in few cases, while Piwil2 and Piwil4 were detected in most of the cases. Moreover, Piwil2 expression was not exclusive to HRS cells but was also detected in most of the microenvironment cells, indicating that the secondary pathway is not exclusive to tumor cells. Along these same lines, *PIWIL2* and *PIWIL4* have been detected in other tumors as well in their normal counterpart [36, 41].

To further explore the activity of the Piwi/piRNA pathway in cHL, we investigated if three piRNAs (piR-651, piR-20365, piR-20582) that had previously been identified as overexpressed in solid tumors [25, 26] could be detected in cHL. The three piRNAs were detected in all the samples, including lymph nodes from cHL patients, reactive lymph nodes used as controls, and the four cHL cell lines. The detection of the piRNAs in control samples indicated that probably a piRNA pool is maintained in somatic tissues [32, 33], including lymph nodes.

In fact, the study by in situ hybridization of piR-651 showed that it was expressed not only in HRS cells but also in normal follicles, especially at centroblasts. Interestingly, piR-651 expression in patient lymph nodes was significantly higher than in RLN suggesting that this upregulation is due to the presence of the population of HRS cells, which are expressing piR-651 and at the same time producing changes in the surrounding cells that lead to an increase of global piR-651 levels in the HL lymph node.

As previously found in other tumors [25, 26], the three piRNAs were overexpressed in the tumor samples.

We then examined the potential correlation of the expression of these piRNAs in lymph node samples with patient outcome and found that piR-651 emerged as an independent prognostic marker for DFS and OS. The patients with low levels of piR-651 had worse outcome. Remarkably, patients that did not achieve treatment response had lower levels of piR-651.

In the same line, the analysis of piR-651 in serum samples showed that piR-651 levels were downregulated at diagnosis but increased after treatment when the patient achieved CR. Cui et al. showed that piR-651 analyzed in mononuclear cells from peripheral blood from gastric cancer patients was also lower in comparison with control samples [42]. This leads us to speculate that the expression of piR-651 detected in serum could come from circulating cells rather than from tumor cells and the downregulation observed in the patients could reflect differences in the peripheral blood populations associated to the presence of the lymphoma. It has previously been reported that immune suppression associated with the lymphoma pathogenesis may be found systemically resulting from an altered monocyte phenotype in patients with lymphoma [43].

Similar results have been observed when studying serum expression of microRNAs in other tumors, including multiple myeloma, where several miRNAs were underexpressed in serum at diagnosis but increased at CR, and lower levels in serum were associated with shorter progression-free survival [44]. In cHL, only one previous study has examined microRNAs in plasma samples, and the authors identified several microRNAs, including miR-494 and miR-1973, whose levels were upregulated at diagnosis and decreased at CR [8]. This is in line with our results and indicates that the analysis of small non-coding RNAs in serum/plasma samples could be useful to monitor treatment response.

Contrary to our results, piR-651 was identified as an oncogene in gastric cancer, where Cheng and coworkers demonstrated *in vitro* that the inhibition of piR-651 was associated with a decrease in cell growth due to an arrest in G2/M phase of gastric cancer cell lines [25]. In contrast, though we have also observed overexpression of piR-651 in patient samples in comparison with RLN, low levels of piR-651 were associated with worse patient outcome. It has previously been demonstrated that other small non-coding RNAs, such as microRNAs, can play a dual role as an oncogene or a tumor suppressor gene according to the cellular context [45]. Treatment has been shown to produce changes in the cellular context affecting the function of some microRNAs, which become oncogenes or tumor suppressor genes when treatment is administered by a target-dependent mechanism [46]. Like microRNAs, piRNAs are involved in post-transcriptional regulation through a similar mechanism based on targeting RNAs by base pairing between complementary sequences that leads to translational repression [23]. To date, only a few studies have associated piRNAs and cancer, most of which have shown that piRNAs are overexpressed and act as oncogenes [25–29, 41, 47]. However, piRNAs can also play a dual role depending on the tumor. For example, piR-823 has been shown to act as a tumor suppressor in gastric cancer [29] and as an oncogene in multiple myeloma [28].

In summary, we show here for the first time that the Piwi/piRNA pathway is active in cHL. Moreover, we have identified a piR-651 as a potential biomarker that can be detected in serum. piR-651 may act as a tumor suppressor since patients with low piR-651 levels had shorter OS, associated with worse response to first-line treatment. Interestingly, piR-651 levels in serum increase at response to treatment. Further investigation in a larger cohort of patients is warranted to confirm these findings and to further validate the potential importance of piR-651 as a prognostic marker in cHL.

## MATERIALS AND METHODS

### Patients

Ninety-four patients diagnosed with cHL at a single institution between February 1994 and December 2010 were included in the analysis. The only exclusion criterion was HIV+. Median age was 38 years, and 48% were male. The most frequent histological subtypes were nodular sclerosis (65%) and mixed cellularity (19%). Using criteria previously described [7], we found that Epstein-Barr virus was present in 30% of the samples. The most frequent first-line treatments were ABVD (62%) and MOPPABV (29%). After first-line treatment, 76 patients (81%) achieved complete remission, two (2%) partial remission, and 11 (11.7%) treatment failure, while five patients (5.3%) died before response evaluation (Table 2). With a median follow-up of 133.5 months (range, 2.8-244.1 months), overall survival was 78.7%. Twelve reactive lymph nodes were used as controls. Serum samples from eleven prospectively collected cHL patients at diagnosis and nine paired samples at complete response and from 10 healthy controls were used to study the expression of piRNAs in serum. Approval for this study was obtained from the Institutional Review Board of Hospital Clinic, Barcelona. Informed consent was obtained in accordance with the Declaration of Helsinki.

**Table 2:** Demographic and clinical characteristics of cHL patients (N=94)

Variable	N (%)
Median age, yrs (range)	34 (15–89)
≤45	68 (72.3)
>45	26 (27.7)
Male sex	45 (47.9)
Histologic subtype	
Nodular sclerosis	61 (64.9)
Mixed cellularity	18 (19.2)
Lymphocyte-rich	6 (6.4)
Lymphocyte-depleted	2 (2.1)
Not classifiable	7 (7.4)
Stage	
I	6 (6.4)
II	51 (54.3)
III	18 (19.1)
IV	19 (20.2)
Presence of B symptoms	38 (40.4)
Presence of Bulky mass	21 (22.3)
Hemoglobin <105 g/L	19 (20.2)
Albumin <40g/L	54 (57.4)
White-cell count > 15,000/mm^3^	13 (13.8)
Lymphocyte count <600/mm^3^ or <8%	11 (11.7)
EBV	
Positive	28 (29.8)
Negative	49 (52.1)
Unknown	17 (18.1)
First-line treatment	
ABVD^1^	58 (61.7)
MOPPABV^2^	27 (28.7)
MOPP^3^	4 (4.3)
Other	5 (5.3)

1 Twenty patients received ABVD with radiation;

2 Fourteen patients received MOPPABV with radiation;

3 One patient received MOPP with radiation.

### Cell culture

Four cHL cell lines were used: L-428, L-1236, L-540 and HDLM2 (DSMZ - the German Resource Centre for Biological Material). The cell lines used were obtained from DSMZ at the beginning of the current work and tested for mycoplasma contamination at least once time at month. The L-428 and L-1236 cell lines were cultured in RPMI 1640 containing 10% fetal calf serum (Invitrogen, Paisley, UK); the L-540 and HDLM2 cell lines were cultured in RPMI 1640 containing 20% fetal bovine serum (Invitrogen).

### RNA extraction

Total RNA was obtained from formalin-fixed paraffin-embedded (FFPE) lymph nodes as previously described [6, 7] using RecoverAll Total Nucleic Acid Isolation Kit (Life Technologies, Foster City, CA). All patient lymph nodes were obtained at diagnosis. Total RNA from cHL cell lines was extracted using Trizol (Life Technologies) according to the manufacturer's protocol. RNA concentration was obtained using NanoDrop ND-1000 Spectrophotometer (Fisher Scientific, Madrid, Spain).

### mRNA quantification

cDNA was synthesized from total RNA using the High Capacity cDNA Reverse Transcription Kit (Life Technologies) as per the manufacturer's protocol. TaqMan expression assays to determine mRNA levels of *PIWIL1* (Hs01041737_m1), *PIWIL2* (Hs00216263_m1) and *PIWIL4* (Hs00381509_m1) were supplied by Life Technologies. β-actin was used as housekeeping gene. RT-QPCR was performed in a total volume of 20 μl in the ABI Prism 7500 Real Time PCR System (Life Technologies). All samples for each gene were run in triplicate for 40 cycles using the following master mix and thermal cycler conditions: 10 μl of the TaqMan universal PCR master mix, 1 μl of the primers and probes, 2 μl of the cDNA and 7 μl of the RNAse-free water; about 2 min 50°C, 10 min 95°C, 15 s 95°C and 1 min 60°C. Fluorescent emission data was captured, and mRNA concentrations were quantified by using the critical threshold value and 2^−ΔΔCt^ method.

### Western blot

Western Blot analysis was performed as previously described [48] using the following primary antibodies: PIWIL1 (ab85125; Abcam, Cambridge, UK), PIWIL2 (ab98852; Abcam) and PIWIL4 (ab111714; Abcam) and α-tubulin (A9044; Sigma).

### Immunohistochemistry

Five-μm-thick transverse sections of FFPE tissues were serially cut and mounted onto Dako Silanized Slides (Dako, Glostrup, Denmark). The immunohistochemical assay was performed as previously described [49] using rabbit polyclonal antibodies anti-human PIWIL1 (ab85125; Abcam), PIWIL2 (ab98852; Abcam) and PIWIL4 (NBP1-83491; Novus Biologicals, Littleton, CO, USA).

### piRNA expression analysis

To quantify piRNA expression, we made cDNA using miScript II RT Kit (Qiagen) as follows: 2 μl of 5x miScript HiSpec Buffer, 1 μl of 10x miScript Nucleics Mix, 1 μl of miScript Rerverse Transcriptase Mix and 250 ng of RNA in 6 μl of distilled water. This was incubated for 1 hour at 37° and 5 minutes at 95°. The cDNA was then used for RT-QPCR quantification of piRNA expression using miScript SYBR Green PCR Kit (Qiagen). For the RT-QPCR, the universal primer included in the kit was used together with the following primers: piR-651 (5′-AGAGAGGGGCCCGTGCCTTG-3′) [25], piR-20365 (5′-GGCCGTGATCGTATAGTGGTTAGT-3′), piR-20582 (5′-GGTGTAATGGTTAGCACTCTG-3′). RT-QPCR was performed on 7500 Real time PCR (Applied Biosytems). For each sample: 5 μl of 2x QuantiTect SYBR Green PCR Master Mix, 1 μl of 10x miScript Universal Primer, 1 μl of the specific primer (10nM), 2 μl of distilled water and 1 μl of our cDNA template. PCR conditions were: first 15 minutes at 95°C, followed by 40 cycles of 15s at 94°C, 30s at 55°C and 34s at 70°C, and finally the dissociation curve analysis. RNU6B (Hs_RNU6-2_11, Qiagen miScript Primer Assays) was used as endogenous control.

### PiRNA expression analysis in serum samples

Total RNA from serum samples was obtained as previously described [44] using miRNeasy Mini Kit (Qiagen). The quantification of piR-651 levels were performed using the same methodology than in lymph nodes (previous section) but for cDNA synthesis only 100ng of total RNA was used.

### Chromogenic *in situ* hybridization

Custom miRCURY LNA Detection Probe (Exiqon A/S, Vedbaek, Denmark) 5′ fluorescein labelled for piR-651 was used in FFPE tissue sections on Dako Silanized Slides (S·3003; Dako). Chromogenic *in situ* hybridization was performed manually. For dewaxing and antigen retrieval, the sections were manually immersed in Target Retrieval solution, high pH (Dako) and heated in a water bath at 95–99°C for 20 min (Dako PT *Link)*. Slides were pretreated with protease 1 for 15 minutes at 37°C. A total amount of 70μl of 25-nM probe was hybridized in 1X sodium chloride–sodium citrate hybridization buffer (SSC) (Innogenetics, Antwerp, Belgium) up to 52°C for 2 hours. We used a prediluted mouse anti-FITC antibody (Leica Biosystems, NuΔloch, Alemania) for 60 minutes. Immunoperoxidase staining was performed using Advance system/HRP (Dako) and Liquid DAB+ (Dako). Finally, sections were stained with hematoxylin.

### Statistical analyses

We analyzed the association of piRNA expression with disease-free survival (DFS), overall survival (OS) and complete response (CR). DFS was measured from the time of occurrence of a disease-free state or attainment of a CR to disease recurrence or death as a result of lymphoma or acute toxicity of treatment. OS was calculated from the time of diagnosis to the date of death from any cause or last follow-up. Optimal cut-offs of piRNA expression data for DFS and OS were assessed by means of maximally selected log-rank statistics using the Maxstat package (R statistical package, v. 2.8.1, Vienna, Austria) [50]. MaxStat identified a significant cutoff only for piR-651(cutoff=0.25; mean expression of low group:-0.046 [range:-0.37-0.18]; mean expression of high group: 1.27 [range: 0.26-6.11]).

DFS and OS were estimated with the Kaplan-Meier method and compared with the log-rank test. A multivariate regression analysis assessing the significance of individual clinical factors included in the International Prognostic Score and significant piRNAs was performed by using the Cox proportional hazards model with backward selection. The Chi-squared or Fisher's exact test was used to estimate differences in distributions. The multivariate analysis for treatment response was performed by using Binary Logistic regression. All statistical analyses were performed using PAS W Statistics 18 (SPSS Inc., Chicago, IL) and R v2.8.1. The level of significance was set at ≤0.05. All performed tests were two-sided.

## SUPPLEMENTARY FIGURE


