# Supplementary material for: PiwiRNA-651 as marker of treatment response and survival in classical Hodgkin lymphoma
Source: Oncotarget. 2016 Jun 14;7(29):46002–13. doi: 10.18632/oncotarget.10015 (PMC5216777; doi:10.18632/oncotarget.10015)
Supplement: Supplementary file 1 [file oncotarget-07-46002-s001.pdf]

PiwiRNA-651 as marker of treatment response and survival in classical Hodgkin lymphoma

SUPPLEMENTARY FIGURE

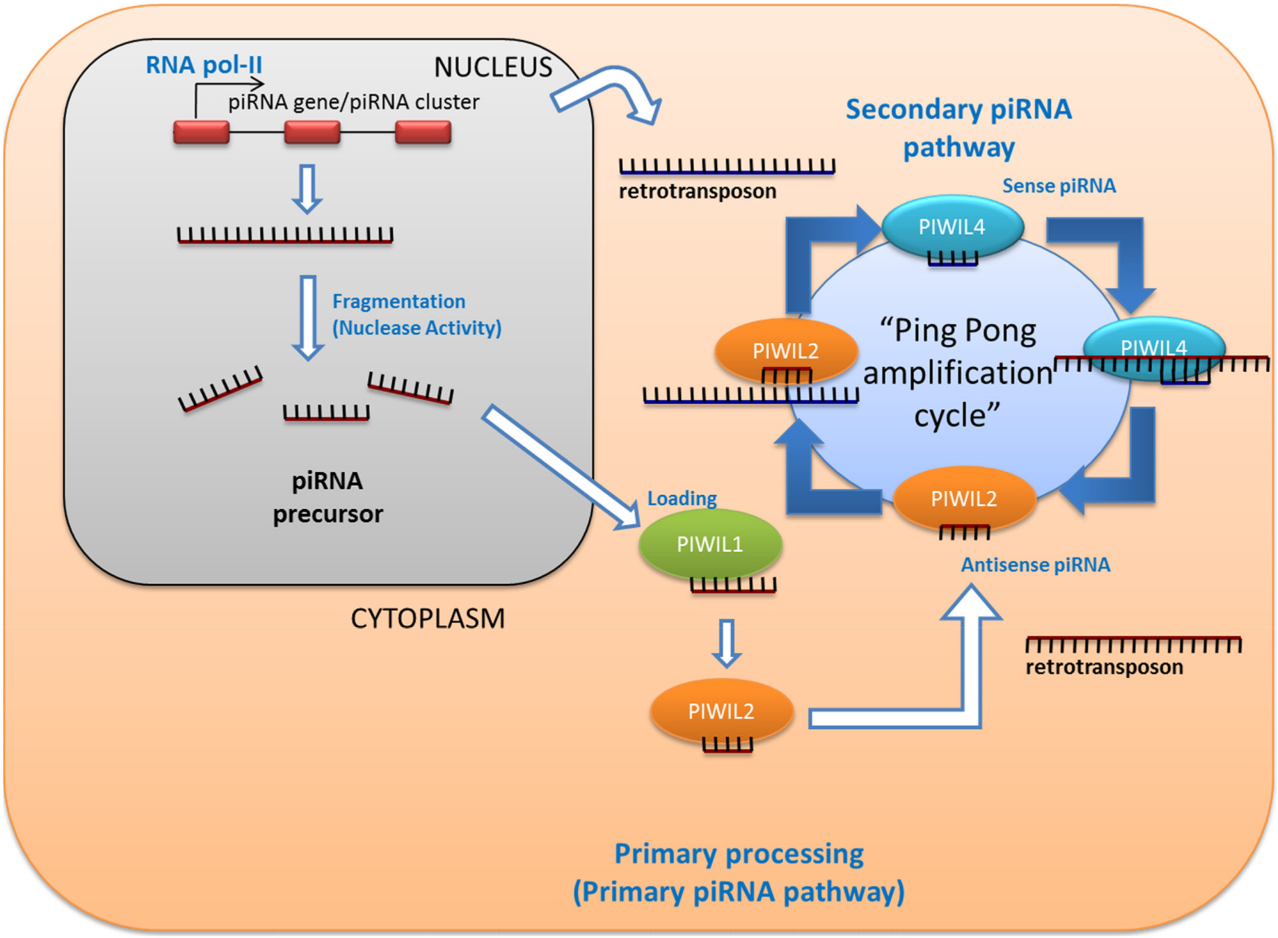

Supplementary Figure S1: piRNA biogenesis pathway.
